# A convolutional neural network-based framework for quality control through speckle displacement analysis

**DOI:** 10.1038/s41598-025-25489-0

**Published:** 2025-11-10

**Authors:** Hamed Sabahno, Davood Khodadad

**Affiliations:** Department of Applied Physics and Electronics, Umeå University, Umeå, 90187 Sweden

**Keywords:** Convolutional neural networks, Quality control, Optical measurement, Speckle pattern analysis, Monte Carlo simulation, Engineering, Mathematics and computing, Optics and photonics

## Abstract

Among the most advanced techniques for quality control, image processing and optical methods are prominent because of their precision and versatility. These methods often involve analyzing speckles generated by coherent laser illumination because coherent light provides detailed and accurate measurement capabilities. In speckle metrology-based techniques, the accurate measurement of speckle displacements is crucial for detecting faults or deformations in objects. In this study, an advanced algorithm segments the image into overlapping grids, followed by a Fourier-based image registration to accurately quantify the speckle displacements. This method can simultaneously detect multiple translational movements in the different parts of an object. However, proper calculation and assignment of overlap sizes to each grid plays a crucial role in this method, which is where we obtain help from convolutional neural networks (CNNs). We develop a CNN architecture and optimize its hyperparameters using a Monte Carlo simulation algorithm incorporating a grid search and *k*-fold cross-validation. Finally, we validate the developed method through a case study involving a simulation and real speckle patterns generated by spraying water on a cardboard surface.

## Introduction

Quality control (QC) is a comprehensive process designed to ensure that products and services adhere to predefined standards and specifications. It involves the continuous monitoring and evaluation of production or service processes to detect and rectify deviations, thereby guaranteeing product consistency, reliability, and customer satisfaction. QC methods include inspection, testing, statistical analysis, and process improvement strategies such as Six Sigma and Total Quality Management (TQM)^1^. In the realm of quality control, image processing and optical techniques are invaluable, offering non-destructive and high-resolution means for assessing product quality and detecting defects. Techniques such as digital image analysis, machine vision, and pattern recognition are utilized to analyze product images and identify deviations from desired standards^2^. Optical methods, including spectroscopy and laser-based imaging, such as interferometry, are employed to measure the surface characteristics and dimensional accuracy with precision^3^. These approaches enable real-time inspection and comprehensive quality assessment across various industries such as manufacturing, healthcare, and electronics.

Speckles (random granular patterns of light resulting from the interference of coherent light scattered by rough or irregular surfaces) play a significant role in non-destructive testing (NDT) and optical metrology^4–6^. Speckle analysis, which involves measuring displacements in speckle patterns, is crucial for quantifying physical changes such as deformation, strain, and flow velocity. Speckle analysis has been used in various applications and in many industries. Some of these applications include non-destructive testing (NDT)^7–10^, optics and metrology^11–19^, medical imaging and diagnostics^20–23^, material and surface science^24^, fluid mechanics^25,26^, and agriculture^27,28^.

Advances in digital speckle interferometry and computational algorithms have enhanced the resolution and accuracy of speckle displacement measurements. Speckle displacement analysis includes various types such as translation, rotation, deformation, shear, surface changes, and optical changes, each affecting the speckle pattern in distinct ways^6,29^. Understanding these displacements helps in interpreting the underlying physical phenomena of the observed system^30–39^.

Image registration, the process of aligning multiple images of the same scene or object taken at different times or from different perspectives, is essential for accurate analysis in QC. This process ensures that images are precisely aligned for effective comparison and detection of deformations. Common image registration methods include intensity-based, feature-based, model-based, transform-based, and frequency domain techniques^40^. Fourier-based image correlation, a robust method in the frequency domain, aligns the images through translation or similarity models. Tong et al.^41^ provided an extensive overview of Fourier-based image registration techniques, categorizing them into intensity-based and feature-based methods.

Convolutional Neural Networks (CNNs) have revolutionized image processing by enabling advanced feature extraction and pattern recognition capabilities. CNNs are particularly effective because of their hierarchical architecture, which leverages convolutional layers to automatically learn the spatial hierarchies of features from raw image data. This ability has led to significant improvements in various image-processing tasks, including object detection, image segmentation, and enhancement^42–48^. CNNs have been applied to enhance speckle pattern analysis and interpretation in speckle metrology. Recent research has highlighted their effectiveness in automating the detection and quantification of speckle patterns and improving the accuracy of displacement and deformation measurements. For instance, CNNs have been employed to refine speckle pattern reconstruction and extract meaningful features from complex interference patterns, thereby advancing non-destructive testing methods and metrological applications. Techniques such as deep learning-based image segmentation and feature extraction have been particularly beneficial in analyzing speckle images, facilitating more precise and reliable measurements in speckle metrology^49–52^.

Recently, Sabahno et al.^53^ introduced an innovative Fourier-based image-registration algorithm tailored for analyzing speckle displacements. In addition, in a letter, Khodadad and Sabahno^26^ used its main concept and showed that speckle’s displacement analysis can be used for fruit quality control. This approach is a scaled and subsectional-based phase correlation that differs from digital image correlation and conventional phase correlation and utilizes a local region-based method in which the image is divided into overlapping grids. Each grid is independently registered to effectively handle local variations and misalignments. However, this approach requires proper determination of the overlapping between each grid individually to effectively measure the displacement of each grid. This paper presents a detailed implementation of a Convolutional Neural Network (CNN) algorithm optimized through a grid search and *k*-fold cross-validation. We use a deep CNN to intelligently determine the appropriate overlap size for each grid. We subsequently apply this algorithm to detect speckle displacements in sequential images of a water-sprayed cardboard. Table 1 highlights the main differences of our proposed CNN-based framework compared to notable available schemes.

**Table 1:** Main differences of the proposed method.

Feature / Method	Full-field phase correlation ^18^	Fixed- grid^19^	Proposed CNN-based framework
Principle	Whole-image FFT registration	Uniform grids	Flexible overlapping grids via CNN
Multiple displacements	Limited	Robust	Robust
Accuracy (complex cases such as high dynamic-range motion)	Poor	Keep trade off	Robust
Efficiency	Very fast	Fast to run; slow to tune	Costly training; fast to run
Key innovation	Noise-robust global shift	Enables local analysis	Learns optimal grid overlaps locally

The paper is organized as follows. Section 1 includes an introduction, important contributions so far, and of course our contribution. Section 2 details the speckle displacement detection and measurement algorithm, and its theoretical basis. Section 3 presents the developed CNN framework. Section 4 presents the experimental setup and simulation studies, and Sect. 5 concludes the paper with remarks and the main results.

## A grid based image registration algorithm

In this section, we describe a grid-based algorithm designed to account for overlapping in detecting variations in speckle movement, both in size and direction, across different regions of the speckle pattern. The algorithm divides the image into grids and utilizes overlapping to improve the movement detection within each grid. This method is essential for simultaneously identifying diverse movements in various parts of a speckle pattern. The accuracy of the algorithm is significantly affected by the grid size and the extent of overlapping. First, we explain the operational mechanics of the algorithm, followed by an exploration of the underlying mathematical theory.

The algorithm for detecting and measuring speckle displacements in an image follows the following steps:

Step 1: Loading images

Load the images to be analyzed. One is taken when there is no deformation in the object (reference image) and the other is taken later for comparison with the reference image.

Step 2: Defining grids size

Divide each image into an equal-sized grid network.

Step 3: Defining grids overlaps

Determine the overlap size between adjacent grids according to a specified overlap percentage (0 or greater). The overlap size is computed as $\:\text{f}\text{l}\text{o}\text{o}\text{r}(\text{g}\text{r}\text{i}\text{d}\:\text{s}\text{i}\text{z}\text{e}\:\times\:\:\text{o}\text{v}\text{e}\text{r}\text{l}\text{a}\text{p}\:\text{p}\text{e}\text{r}\text{c}\text{e}\text{n}\text{t}\text{a}\text{g}\text{e}/100)$. The overlap percentage can be equal for all the grids, or each grid can have a different overlap size.

Step 4: Registration loops

Iterate through each grid, extract the corresponding areas from both images, and individually perform image registration for each grid.

Step 5: Calculating displacements

Extract displacement vectors from the transformation matrix and store them.

Step 6: Visualization

Plot the grid centers and displacement arrows on the reference image.

In what follows, we explain the theory behind Step 4 (image registration), and Step 5 (displacements calculation).

The image registration is carried out using the translation registration technique. Following the method described by Reddy and Chatterji^18^, a phase correlation is employed to estimate the translation vector between the two grids of the images. This involves computing the cross-power spectrum of the Fourier transforms of the grids and identifying the peak in the resulting correlation matrix. By transforming the spatial domain grids into the frequency domain, phase correlation techniques are used to determine translation shifts. The principle behind phase correlation is that translation in the spatial domain translates into a linear phase shift in the frequency domain^54^.

To address spectral leakage, which can occur owing to the periodic extension of grids during Fourier transformation, windowing is applied. Spectral leakage can introduce artifacts that affect the translation detection accuracy. The Blackman window is particularly effective in reducing these artifacts by smoothly tapering the grid boundaries.

The process of detecting displacements between two grids using the Fourier transform involves:

1. Conversion to the Frequency Domain: Apply the Fourier transform to both grids, converting them from spatial intensity to spatial frequencies. This change represents the grids in terms of their spatial frequencies rather than pixel intensities.
2. Phase Correlation: In the frequency domain, translation appears as a linear phase difference. Compute the cross-power spectrum of the Fourier-transformed grids and then perform the inverse Fourier transform of this cross-power spectrum. The result is a correlation matrix in the spatial domain, with the peak indicating the translation offset between the grids.
3. Translation Detection: Analyze the peak of the correlation matrix to determine the translation vector that aligns the two grids. This vector indicates the displacement between the grids in terms of the horizontal and vertical shifts.

As mentioned previously, prior to applying the Fourier transform, windowing with a Blackman window is used to reduce spectral leakage and enhance the stability of the registration results^55^. This step is crucial when using FFT (Fast Fourier Transform) to avoid artifacts caused by abrupt transitions at grid edges.

1 $$\:w\left(z\right)={a}_{0}-{a}_{1}\text{cos}\left(\frac{2\pi\:z}{T-1}\right)+{a}_{2}\text{c}\text{o}\text{s}\left(\frac{4\pi\:z}{T-1}\right),$$

where $\:w\left(z\right)$ is the value of the window function at index *z*, *z* is the index along one of the rows or columns of the section, *T* is the total number of samples in the window, $\:{a}_{0}=0.42$,$\:{a}_{1}=0.5$, and $\:{a}_{2}=0.08$.

This formula generates the coefficients of the Blackman window, which are then multiplied element-wise with $\:I\left(x,y\right)$, the intensity of the grids at the spatial coordinates of (*x*,* y*), to apply the windowing effect. Therefore, we have:

2 $$\:{f}_{1}\left(x,y\right)={I}_{1}\left(x,y\right)\times\:w\left(x\right)\times\:w\left(y\right),$$

3 $$\:{f}_{2}\left(x,y\right)={I}_{2}\left(x,y\right)\times\:w\left(x\right)\times\:w\left(y\right),$$

where $\:{f}_{1}\left(x,y\right)$ and $\:{f}_{2}\left(x,y\right)$ are the intensity functions of the fixed (reference) and deformed (moving) grids, respectively, affected by the Blackman window.

The Fourier transform of the spatial domain grid $\:f\left(x,y\right)$ is:

4 $$\:F\left(u,v\right)={\iint\:}_{-\infty\:}^{\infty\:}f\left(x,y\right)\times\:{e}^{-2\pi\:i(ux+vy)}dxdy,$$

where $\:F\left(u,v\right)$ is the Fourier transform of the grid and *u* and *v* are the spatial frequency coordinates.

After performing the Fourier transform, high-pass emphasis filtering is applied to enhance the high-frequency components in the Fourier magnitude spectra while suppressing low-frequency components. This technique improves the robustness of phase correlation by emphasizing finer image details and minimizing the impact of low-frequency background variations.

To achieve this, we first obtain the Fourier magnitude spectra $\:{F}_{1}$ and $\:{F}_{2}$ of the reference and moving grids, respectively, and apply a high-pass filter *H*$\:\left(u,v\right)$ to enhance the high-frequency components. The enhanced Fourier magnitude spectra of the fixed and moving grids become:

5 $$\:{F{\prime\:}}_{1}\left(u,v\right)=\left|{F}_{1}\left(u,v\right)\right|\times\:H\left(u,v\right),$$

6 $$\:{F{\prime\:}}_{2}\left(u,v\right)=\left|{F}_{2}\left(u,v\right)\right|\times\:H\left(u,v\right),$$

where *H*$\:\left(u,v\right)$=(1 − *X*$\:\left(u,v\right)$)(2 − *X*$\:\left(u,v\right)$) and *X*$\:\left(u,v\right)$*=cos*$\:\left(\pi\:u\right).cos\left(\pi\:v\right)$.

The cross-power spectrum (*P*) is then calculated as the complex conjugate of the Fourier transform of one grid multiplied by the Fourier transform of the other grid:

7 $$\:P\left(u,v\right)=\frac{{F{\prime\:}}_{1}\left(u,v\right)\times\:{F}_{2}^{{\prime\:}*}\left(u,v\right)}{\left|{F{\prime\:}}_{1}\left(u,v\right)\times\:{F}_{2}^{{\prime\:}*}\left(u,v\right)\right|},$$

where $\:{F}_{2}^{{\prime\:}*}\left(u,v\right)$ denotes the complex conjugate of $\:{F{\prime\:}}_{2}\left(u,v\right)$.

The phase correlation operation involves taking the inverse Fourier Transform of the cross-power spectrum $\:P\left(u,v\right)$ to obtain the correlation matrix *C*(*x*,* y*) in the spatial domain.

8 $$\:C\left(x,y\right)=\text{I}\text{F}\text{F}\text{T}\left\{P\left(u,v\right)\right\},$$

where *C*(*x*,* y*) is the correlation matrix representing the spatial domain and $\:\text{I}\text{F}\text{F}\text{T}$ denotes the inverse fast Fourier transform.

The displacement between the two grids is identified by locating the peak in the correlation matrix, *C*(*x*, *y*). The coordinates of this peak indicate the horizontal and vertical shifts that are required to align the grids.

The cross-power spectrum captures the spatial frequency information, and by analyzing its inverse Fourier transform, we obtain a correlation matrix that reveals the displacement between the grids. The peak of this correlation matrix indicates the optimal displacement values $\:\left(\varDelta\:x,\varDelta\:y\right)$

9 $$\:\left(\varDelta\:x,\varDelta\:y\right)={\text{a}\text{r}\text{g}\:\text{m}\text{a}\text{x}}_{(x,y)}C\left(x,y\right),$$

where $\:\left(\varDelta\:x,\varDelta\:y\right)$ are the detected displacements.

## A convolutional neural network framework

In this section, we describe the development of the CNN framework. We preprocess the images, divide them into sections, extract features, and optimize hyperparameters for the CNN using a comprehensive grid search and *k*-fold cross-validation. The goal is to classify the image grids based on the manually defined overlap percentages. In fact, this CNN framework is supposed to perform Step 3 of the algorithm in Sect. 2, to find and dedicate the appropriate overlap size to each grid, which is the crucial part of that algorithm for its next steps. It is important to note that the grid size is a fixed input parameter, while the overlap percentage is the variable optimized by the CNN for each grid.

The optimization of CNN hyperparameters is crucial for achieving a high performance in image classification tasks. This paper outlines a method for optimizing CNN hyperparameters, including the learning rate, mini-batch size, number of epochs, number of filters, dropout rate, weight decay, and optimizer type, through a systematic grid search and *k*-fold cross-validation approach.

The proposed CNN algorithm works as follows:

Step 1. Image loading and pre-processing

Two pairs of images, $\:{I}_{1}\left(x,y\right)$ and $\:{I}_{2}\left(x,y\right)$, are loaded and converted to double-precision images for processing.

Step 2. Gridding the images

The images are divided into equal-sized grids in each direction, and with *n* grids in each direction, we would have a total of $\:n\times\:n={n}^{2}$ grids in the image. The grid size is calculated to evenly fit into the image dimensions.

Step 3. Defining manual overlaps

In a known dataset, manual overlaps are assigned to each grid to serve as labels for the classification task. The set of manual overlaps is $\:O=[{o}_{1},{o}_{2},{o}_{3},\dots\:,{o}_{{n}^{2}}]$, where $\:{n}^{2}$ is the total number of grids in the image.

Step 4. Preparing data for CNN

Image grids are extracted and resized to match the CNN input size. Grids from both images are stored as the input data, *D*, and the manual overlaps *O* are converted to categorical labels. $\:D=\left\{\left({{I}_{1}}_{ij},{{I}_{2}}_{ij}\right)|i=\text{1,2},\dots\:n,\:\:j=\text{1,2},\dots\:n\:\right\}$, where $\:{{I}_{1}}_{ij}$ and $\:{{I}_{2}}_{ij}$ are the corresponding grids in images 1 (reference image) and 2 (test image).

Step 5. Defining and calculating class weights

The class weights are calculated to handle the class imbalance in the training data. The class weights for each class are calculated as follows:

10 $$\:{w}_{c}=\frac{N}{C\times\:{n}_{c}},$$

where $\:{w}_{c}$​ is the weight for class *c*, *N* is the total number of samples (total number of grids, which in our case *N=*$\:{n}^{2}$), *C* is the total number of classes, and $\:{n}_{c}$ is the number of samples (grids) with class *c*.

Step 6. Grid search and k-fold cross-validation

A grid search is implemented to explore a range of hyperparameter values. The hyperparameter sets are $\:{\Phi\:}=[{\varphi\:}_{1},{\varphi\:}_{2},{\varphi\:}_{3},\dots\:,{\varphi\:}_{q}]$, where $\:{\varphi\:}_{i}$ is a specific set of hyperparameter values and *q* is the total number of hyperparameter value sets to be evaluated and explored.

The main hyperparameters in a CNN structure are:

- Learning rate ($\:\eta\:)$: Affects the speed and stability of the convergence of the model.

The learning rate controls the step size at each iteration while moving towards the minimum of the loss function. The gradient descent update rule is:

11 $$\:{\theta\:}_{t+1}={\theta\:}_{t}-\eta\:{\nabla\:}_{\theta\:}J\left({\theta\:}_{t}\right),$$

where $\:\theta\:$ represents the parameters of the model (weights and biases), *t* is the iteration step, $\:\eta\:$ is the learning rate, and $\:{\nabla\:}_{\theta\:}J\left({\theta\:}_{t}\right)$ is the gradient of the loss function *J* with respect to *θ* at *t*.

- Mini-batch size (*B*): Balances the computational efficiency and the quality of the gradient estimate. The mini-batch size is the number of training examples used to calculate the gradient at each iteration.

The batch gradient descent is:

12 $$\:{\nabla\:}_{\theta\:}J\left(\theta\:\right)=\frac{1}{B}\sum\:_{i=1}^{B}{\nabla\:}_{\theta\:}J\left(\theta\:;{x}_{i},{y}_{i}\right),$$

where *B* is the mini-batch size and ($\:{x}_{i},{y}_{i}$) represents the training samples ($\:{x}_{i})$ and their corresponding labels ($\:{y}_{i})$.

- Number of epochs: Determines how long the model trains and how well it fits the data.

The number of epochs is the number of complete passe through the entire training dataset. Each epoch involves one full pass through the entire training dataset, during which the model’s weights are updated incrementally using all training samples, processed in smaller groups called mini-batches.

The number of iterations per epoch is calculated as $\:\frac{\text{N}\text{u}\text{m}\text{b}\text{e}\text{r}\:\text{o}\text{f}\:\text{T}\text{r}\text{a}\text{i}\text{n}\text{i}\text{n}\text{g}\:\text{S}\text{a}\text{m}\text{p}\text{l}\text{e}\text{s}}{B}$.

- Number of filters: Determines the number of features detected in each layer.

Filters (or kernels) are used in the convolutional layers to detect features in the input image.

The convolution Operation is:

13 $$\:\left(I*K\right)\left(i,j\right)=\sum\:_{m=0}^{h-1}\sum\:_{n=0}^{w-1}I\left(i+m,j+n\right)K\left(m,n\right),$$

where *I* is the *H*×*W* input image and *K* is the *h*×*w* kernel/filter applied to the image. The number of filters determines how many such kernels are used in the layer; (*i*,* j*) are the coordinates in the output feature map with *i* from 0 to *H*-*h* and *j* from 0 to *W*-*w*, and (*m*,* n*) are the coordinates within the filter *K*.

- Dropout rate (*p*): Prevents overfitting by introducing noise during training.

Dropout is a regularization technique that prevents overfitting by randomly setting the fraction *p* of the input units to zero at each update during training.

14 $$\:\stackrel{\sim}{h}=\frac{h}{1-p}\times\:r,$$

where *h* is the input to the dropout layer (the activations from the previous layer), *r* is a random binary mask where each element is 1 with probability 1 − *p* and 0 with probability *p*, and $\:\stackrel{\sim}{h}$ is the output after applying dropout.

- Weight decay $\:(\lambda\:$): Regularizes the model by penalizing large weights, preventing overfitting.

Weight decay is a regularization technique that adds a penalty proportional to the magnitude of the weights to the loss function. The regularization term is

15 $$\:{J}_{total}=J\left(\theta\:\right)+\lambda\:{‖\theta\:‖}^{2},$$

where $\:J\left(\theta\:\right)$ is the original loss function and $\:\lambda\:{‖\theta\:‖}^{2}$ is the weight decay term.

- Optimizer type: Affects the efficiency and effectiveness of the training process, handling different challenges in gradient-based optimization.

Common optimizers include Stochastic Gradient Descent (SGD) and Adaptive Moment Estimation (Adam). Adam is built on ideas from two other optimization methods Momentum and RMSProp, combining them in a way that adapts the learning rate for each parameter. In the following, the mechanisms of SGD and Adam are explained.

Stochastic Gradient Descent (SGD):

With SGD, the parameter *θ* is updated for each iteration *t* as shown in Eq. 11. Here, $\:\eta\:$ (learning rate) directly scales the gradient, controlling the size of the step towards the minimum of the loss function.

Adaptive Moment Estimation (Adam):

$$\:{m}_{t}={\beta\:}_{1}{m}_{t-1}+\left(1-{\beta\:}_{1}\right){\nabla\:}_{\theta\:}J\left({\theta\:}_{t}\right),$$

$$\:{v}_{t}={\beta\:}_{2}{v}_{t-1}+\left(1-{\beta\:}_{2}\right){{\nabla\:}_{\theta\:}J\left({\theta\:}_{t}\right)}^{2},$$

$$\:{\widehat{m}}_{t}=\frac{{m}_{t}}{1-{\beta\:}_{1}^{t}},\:{\widehat{v}}_{t}=\frac{{v}_{t}}{1-{\beta\:}_{2}^{t}},$$

16 $$\:{\theta\:}_{t+1}={\theta\:}_{t}-\eta\:\frac{{\widehat{m}}_{t}}{\sqrt{{\widehat{v}}_{t}}+\epsilon\:},$$

where *m* and $\:v$ are estimates of the first and second moments of the gradient; $\:{\beta\:}_{1}$​ (mean of the gradients, typically set to 0.9) and $\:{\beta\:}_{2}$ (uncentered variance of the gradients, typically set to 0.999) are the hyperparameters for these estimates; and $\:\epsilon\:$ is a small constant (typically set to 10^− 8^). In Adam, $\:\eta\:$ scales the ratio of the bias-corrected first moment estimate $\:{\widehat{m}}_{t}$​ to the square root of the bias-corrected second moment estimate $\:{\widehat{v}}_{t}$​, ensuring that updates are well scaled for different parameters.

The *k*-fold cross-validation is a resampling procedure used to evaluate the performance of a machine-learning model. It divides the dataset into *k* subsets (folds) and ensures that each fold is used exactly once as a test set. Given a dataset *D* with *N* samples, we divide *D* into *k* approximately equal-sized folds. This process is repeated *k* times, with each fold used exactly once as the validation set. A *k*-fold cross-validation strategy is used to evaluate each hyperparameter set. The best set is selected based on the average score across the folds.

17 $$\:Score\left({\Phi\:}\right)=\frac{1}{k}\sum\:_{i=1}^{k}evaluate({\Phi\:},{T}_{i},{V}_{i}),$$

where *k* is the number of folds, and $\:{T}_{i}$ and $\:{V}_{i}$​ are the training and validation sets for the fold *i*. The evaluation is performed using an objective function, which in our case we use:

18 $$Objective{\text{ }}Function{\text{ }} = {\text{ }}0.5~F1Score{\text{ }} + {\text{ }}0.5~Accuracy,$$

with *Accuracy*=$\:\frac{\sum\:_{i=1}^{N}1({y}_{i}={\widehat{y}}_{i})}{N}$, where $\:{y}_{i}$ and $\:{\widehat{y}}_{i}$ are the true and predicted labels, respectively; and **1**(.) is an indicator function that returns 1 if the argument is true and 0 otherwise, and *F1Score*=$\:\frac{\sum\:_{c=1}^{C}2\times\:\frac{{Perecision}_{c}\times\:{Recall}_{c}}{{Perecision}_{c}+{Recall}_{c}}}{C}$, where $\:{Perecision}_{c}=\frac{{TP}_{c}}{{TP}_{c}+{FP}_{c}}$ (positive predictive value) and $\:{Recall}_{c}=\frac{{TP}_{c}}{{TP}_{c}+{FN}_{c}}$ (sensitivity or true positive rate) and $\:{TP}_{c}$, $\:{FP}_{c}$, and $\:{FN}_{c}$ are the true positives, false positives, and false negatives for class *c*, respectively.

Step 7. Defining CNN architecture and training with the best hyperparameters

The CNN architecture is defined based on the best hyperparameter set obtained from the previous step. The network is trained using these hyperparameters, and the final model evaluated using the test set. The CNN includes many layers:

$$\:CNN\:Architecture=\left(\begin{array}{c}Input\:Layer\\\:Convolution\:Layer\\\:\begin{array}{c}Batch\:Normalization\:Layer\\\:ReLU\:Layer\\\:\begin{array}{c}\begin{array}{c}Pooling\:Layer\\\:Dropout\:Layer\end{array}\\\:\begin{array}{c}Fully\:Connected\:Layer\\\:Softmax\:Layer\\\:Classification\:Layer\end{array}\end{array}\end{array}\end{array}\right)$$

### Input layer

The input layer ensures that the data fed into the network matches the dimensions expected by the subsequent layers, allowing the CNN to process and learn effectively from the image gids. The input data in our case are four-dimensional and include: [grid height, grid width, channels (which is equal to two), and samples (grids)].

### Convolutional layer

The convolutional layer is the core building block of the CNN. It applies a series of filters (kernels) to the input image to produce the feature maps. Each filter is a small matrix that slides over the image, performing element-wise multiplications and summing the results. This operation is known as a convolution (Eq. 13).

### Batch normalization layer

The batch normalization layer normalizes the activation of the previous layer for each mini-batch. This helps stabilize and accelerate training. Mathematically, if *x* is the input to the batch normalization layer, the output $\:\widehat{x}$ is computed as

19 $$\:\widehat{x}=\frac{x-\mu\:}{\sqrt{{\sigma\:}^{2}+\epsilon\:}}\times\:\gamma\:+\beta\:,$$

where $\:\mu\:$ is the mean of the mini-batch, $\:{\sigma\:}^{2}$ is the variance of the mini-batch, $\:\epsilon\:$ is a small constant for numerical stability, and $\:\gamma\:$ and $\:\beta\:$ are learnable scaling and shifting parameters, respectively.

### Activation function (ReLU Layer)

The activation function introduces nonlinearity into the network, enabling it to learn complex patterns. The Rectified Linear Unit (ReLU) function is commonly used, which is defined as:

20 $$\:ReLU\left(x\right)=\text{m}\text{a}\text{x}(0,x),$$

For each element *x* in the feature map, the ReLU outputs *x* if *x* is positive; otherwise, it outputs 0. This function helps the network to learn non-linear representations.

### Pooling layer (Max pooling)

The pooling layer reduces the spatial dimensions (height and width) of the feature maps while retaining the most important information. Max pooling is a common technique in which the maximum value is obtained from each patch of a feature map.

For a pooling operation with filter size ​ $\:{p}_{h}\times\:{p}_{w}$ and stride *s* (which determines how far the pooling window moves for the next operation), the max pooling operation is defined as:

21 $$\:{P}_{i,j}={max}_{0\le\:m<{p}_{h},0\le\:n<{p}_{w}}\left[F({s}_{i}+m,{s}_{j}+n)\right],$$

where *F* is the input feature map; $\:{P}_{i,j}$ is the pooled (output) feature map at position (*i*,*j*); $\:{s}_{i}$ is the starting row index for the pooling window; and $\:{s}_{j}$ is the starting column index for the pooling window. Note that if stride *s* is the same in both dimensions (as in our case), then: $\:{s}_{i}=s.i$ and $\:{s}_{j}=s.j$.

### Dropout layer

The dropout layer is a crucial component in neural network architectures and is used to prevent overfitting by randomly dropping units during the training.

The output $\:{\stackrel{\sim}{h}}_{i}$ of neuron *i* after applying dropout is given by:

22 $$\:{\stackrel{\sim}{h}}_{i}=\left\{\begin{array}{cc}{h}_{i}/(1-p)&\:if\:{r}_{i}=1\\\:0&\:if\:{r}_{i}=0\end{array}\right.,$$

where $\:{r}_{i}$ is the dropout mask indicating whether the neuron is dropped or not. Note that the retained neurons are then scaled by $\:1/(1-p)$ to compensate for the fact that dropout reduces the number of active neurons.

### Fully connected layer

The fully connected layers (dense layers) connect each neuron in the current layer to every neuron in the previous layer. These are typically used at the end of the network to produce the final output.

For a fully connected layer with input vector *x* and weight matrix *W*, output vector y is computed as

23 $$\:y=W\times\:x+b,$$

where *b* is the bias vector.

### Softmax layer

The Softmax layer converts the raw output scores (logits) of the network into probabilities that sum to one, which represents the likelihood of each class. Given the input vector $\:\varvec{z}=\left[{z}_{1},{z}_{2},\dots\:,{z}_{tc}\right]$, where *tc* is the number of classes, the Softmax function for class *i* is applied as:

24 $$\:{p}_{i}=\frac{{e}^{{z}_{i}}}{{\sum\:}_{j=1}^{tc}{e}^{{z}_{j}}},$$

where $\:{p}_{i}$ is the probability of class *i* and $\:{z}_{i}$ is the raw score (logit) for class *i*. The output in this layer is a vector, where each element represents the probability of a class, that is, $\:\varvec{p}=\left[{p}_{1},{p}_{2},\dots\:,{p}_{C}\right]$.

### Classification layer

The purpose of the classification layer is to compute the classification loss and to evaluate the performance of the network during training and validation. It also handles the final step of converting the network output into a predicted class label. It contains the following three components.

i) Loss function: For a multi-class classification problem, the classification layer typically uses a cross-entropy loss function. For true labels $\:\varvec{y}=({y}_{1},{y}_{2},\dots\:,{y}_{C})$, where $\:{y}_{c}$​ is 1 if class *c* is the true class, and 0 otherwise, and the predicted labels $\:\widehat{\varvec{y}}=({\widehat{y}}_{1},{\widehat{y}}_{2},\dots\:,{\widehat{y}}_{C})$, where $\:{\widehat{y}}_{c}$ is the predicted probability for class *c*, the cross-entropy loss is calculated as $\:L(\widehat{\varvec{y}},\varvec{y})=-\sum\:_{c=1}^{C}{y}_{c}log\left({\widehat{y}}_{c}\right)$.
ii) Class Weights: The classification layer can incorporate class weights if some classes are more important or if there is a class imbalance. The weights adjust the contribution of each class to the loss. Therefore, the cross-entropy loss by considering the class weights is calculated as $\:L(\widehat{\varvec{y}},\varvec{y})=-\sum\:_{c=1}^{C}{{w}_{c}y}_{c}log\left({\widehat{y}}_{c}\right)$.
iii) Output: The classification layer outputs the loss value for a batch of data that is used to update the weights of the netwok during training. It also determines the predicted class by selecting the class with the highest probability from the Softmax layer.

Softmax and classification layers are typically used together at the end of the network to handle the final stage of classification stage. This is how the Softmax and classification layers work together:

Forward Pass:

- The final fully connected layer of the network produces raw scores (logits) for each class.
- The Softmax Layer converts these logits into probabilities.
- The classification layer calculates the loss based on the true class labels and the predicted probabilities from the Softmax layer.

Backward Pass (training):

- During backpropagation, the classification layer computes the gradient of the loss function with respect to the network weights.
- These gradients are then used to update the weights of the network to minimize loss.

## Simulation and case study

In this section, we use a real case to test the proposed method. During an experimental setup, we sprayed water on a cardboard, and shortly after the spray, captured a picture while emitting laser beams on the object and created a reference image (speckle pattern).

**Fig. 1:**
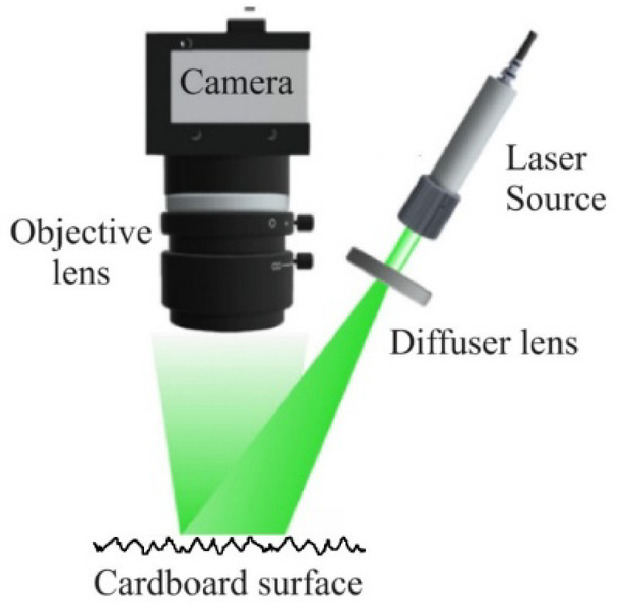
The schematic of the experimental setup.

The experiments were conducted at room temperature (22–25 °C) under standard laboratory conditions, with humidity consistent with the ambient environment. Illumination was provided by a diode-pumped solid-state laser (Thorlabs CPS532-C2; λ = 531.8 nm, *P* = 0.9 mW). The beam was passed through a diffuser to achieve uniform illumination on the cardboard and directed at a slight angle to reduce specular return. Speckle images were captured with a monochrome camera (FLIR BFS-U3-120S4M-CS, Sony IMX226 sensor, 4000 × 3000 pixels, 1.85 μm pixel size) fitted with a Thorlabs MVL8M23 lens (f = 8 mm, f/4). The camera–sample distance was ~ 17 cm; in this configuration, ~ 5.5 cm of the object spans 1500 pixels (see Fig. 1 for reference).

Initially, the surface of the cardboard remained dry. Following this, we applied water randomly to the surface using a fine mist spray bottle, dispersing the droplets in a random pattern. A reference image was captured after spraying.

### Creating the dataset

First, we needed to obtain a dataset, and in the process, we tested the proposed method in Sect. 2. We created local artificial shifts in different areas of the reference speckle pattern and created a second speckle pattern image. We then investigated whether or not our algorithm in Sect. 2 can detect and measure the displacements simultaneously by manually setting a universal overlap percentage for all the image grids. We later used these two speckle patterns (the reference image and the artificially changed image) to train our CNN model.

**Fig. 2:**
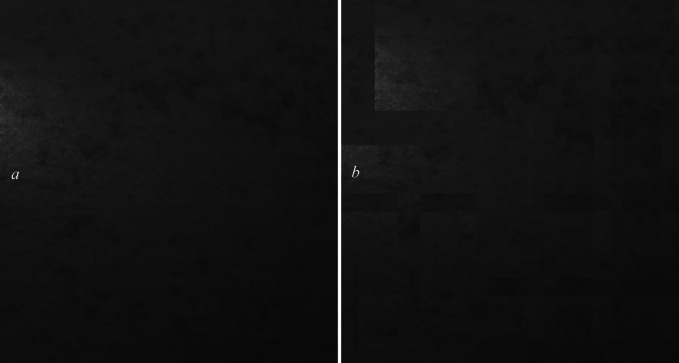
Reference (**a**) and shifted (**b**) speckle patterns.

We cropped the captured images into 1500 pixels X 1500 pixels speckle pattern images. Displacements were applied to the three main areas of the speckle patterns. The speckle patterns before and after the shifts are shown in Fig. 2.

The sizes of the affected areas and shift (displacement) sizes for each area of the image were divided into three main areas.

***Area one***:

Upper-Left Corner:

- Shift in the X direction: 148 pixels to the right.
- Shift in the Y direction: 142 pixels up.
- Affected area size: 600 pixels (width) $\:\times\:$ 600 pixels (height).

***Area two***:

Upper-right corner:

- Shift in the X direction: 20 pixels to the left.
- Shift in the Y direction: 16 pixels down.
- Affected area size: 800 pixels (width) $\:\times\:$ 100 pixels (height).

Center:

- Shift in the X direction: 18 pixels to the left.
- Shift in the Y direction: 18 pixels down.
- Affected area size: 200 pixels (width) $\:\times\:$ 200 pixels (height).

Above the center:

- Shift in the X direction: 18 pixels to the left.
- Shift in the Y direction: 18 pixels down.
- Affected area size: 200 pixels (width) $\:\times\:$ 500 pixels (height).

Bottom-right corner:

- Shift in the X direction: 18 pixels to the right.
- Shift in the Y direction: 18 pixels down.
- Affected area size: 60 pixels (width) $\:\times\:$ 60 pixels (height).

Bottom-left side of the image but not in the corner, after an area of 50 × 50 pixels:

- Shift in the X direction: 25 pixels to the right.
- Shift in the Y direction: 34 pixels up.
- Affected area size: 550 pixels (width) $\:\times\:$ 150 pixels (height).

Above the bottom left area:

- Shift in the X direction: 25 pixels to the right.
- Shift in the Y direction: 34 pixels up.
- Affected area size: 250 pixels (width) $\:\times\:$ 150 pixels (height).

***Area three***:

Other affected areas:

- Shift in the X direction: 80 pixels to the left.
- Shift in the Y direction: 75 pixels down.
- Except for one area that is located at the bottom right of the center and has a dimensions of 300 pixels (width) $\:\times\:$ 250 pixels (height), the others (10 identical areas) have dimensions of 250 pixels (width) $\:\times\:$ 250 pixels (height).

Note that we chose a very complex shifted pattern and also, divided the affected areas into three main areas, so we can find and dedicate an overlap percentage to each area, and better train the CNN structure later on.

We first test our algorithm in Sect. 2 to detect these shifts (displacements) without using artificial intelligence (CNN). As mentioned in the previous section, before running the algorithm, we need to define the grid and overlap sizes, and their values are highly relevant to the size of the affected area and also the shift sizes. If the grid size is noticeably larger or smaller than the affected area or shift sizes, the algorithm may not properly detect the shift with an overlap of zero. However, if a proportional overlap size is chosen, the algorithm can detect shifts. Nonetheless, the proper grid and overlap sizes may be different for different affected areas and shift sizes. In practice, since the size of the affected area and the shift sizes are unknown, we better choose a smaller grid size. Choosing the correct overlap size is another, and more demanding, challenge. The best overlap size might differ for each grid, because the shift sizes and the affected area sizes are different; some need a small overlap, and some need large overlaps to be detected/measured. In this section, we divide the image into 30 equal-sized grids in each direction, *n* = 30, making the total number of grids in the image: *n*^2^ = 900, and the grid sizes are 1500/30 = 50 in both the X and Y directions.

**Fig. 3:**
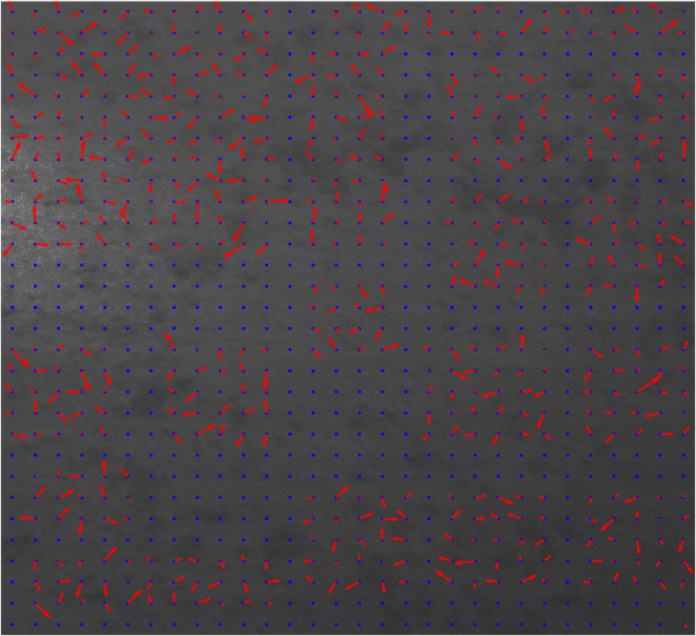
Shifted sections with the corresponding displacement vectors calculated with zero overlaps.

Figure 3 shows the reference image by plotting each grid’s displacement vectors (if any) in red computed using the algorithm outlined in Sect. 2 and setting the overlap size for each grid to zero. The blue points represent the centers of each grid.

As shown in Fig. 3, using the algorithm described in Sect. 2, all the shifted areas can be detected, but almost none of the grid displacements are correctly calculated when we do not let the grids have any overlaps during the execution of the algorithm and use their shared characteristics.

**Fig. 4:**
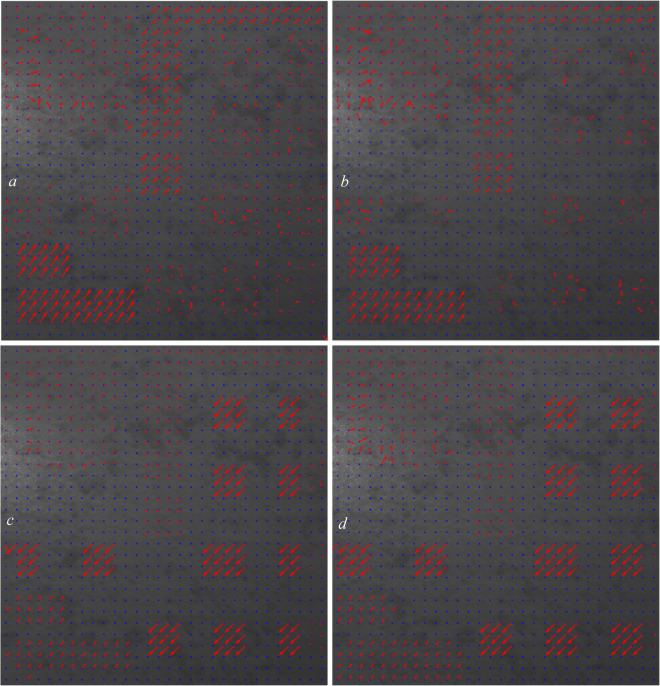
Computed displacement vectors with the overlaps of (**a**): 0.5, (**b**): 1, (**c**):1.5, and (**d**): 2.

In Figure 4, we allow the grids to have overlap. In this figure, we tested the overlap percentages of (0.5, 1, 1.5, 2) $\:\times\:$100.

In this analysis, because we applied the displacements ourselves, we know which overlap percentages suit which grids. Based on the four images plotted in Fig. 4, we can see that the overlap of 0.5 (Fig. 4a) suits the affected area two very well. With this overlap (0.5), the displacements in other grids in other affected areas are not correctly calculated and that would be great when we want to train the CNN structure and use this dataset as our training dataset because we can manually dedicate the overlap percentage of 0.5$\:\times\:$100 = 50% for the grids in this area.

**Fig. 5:**
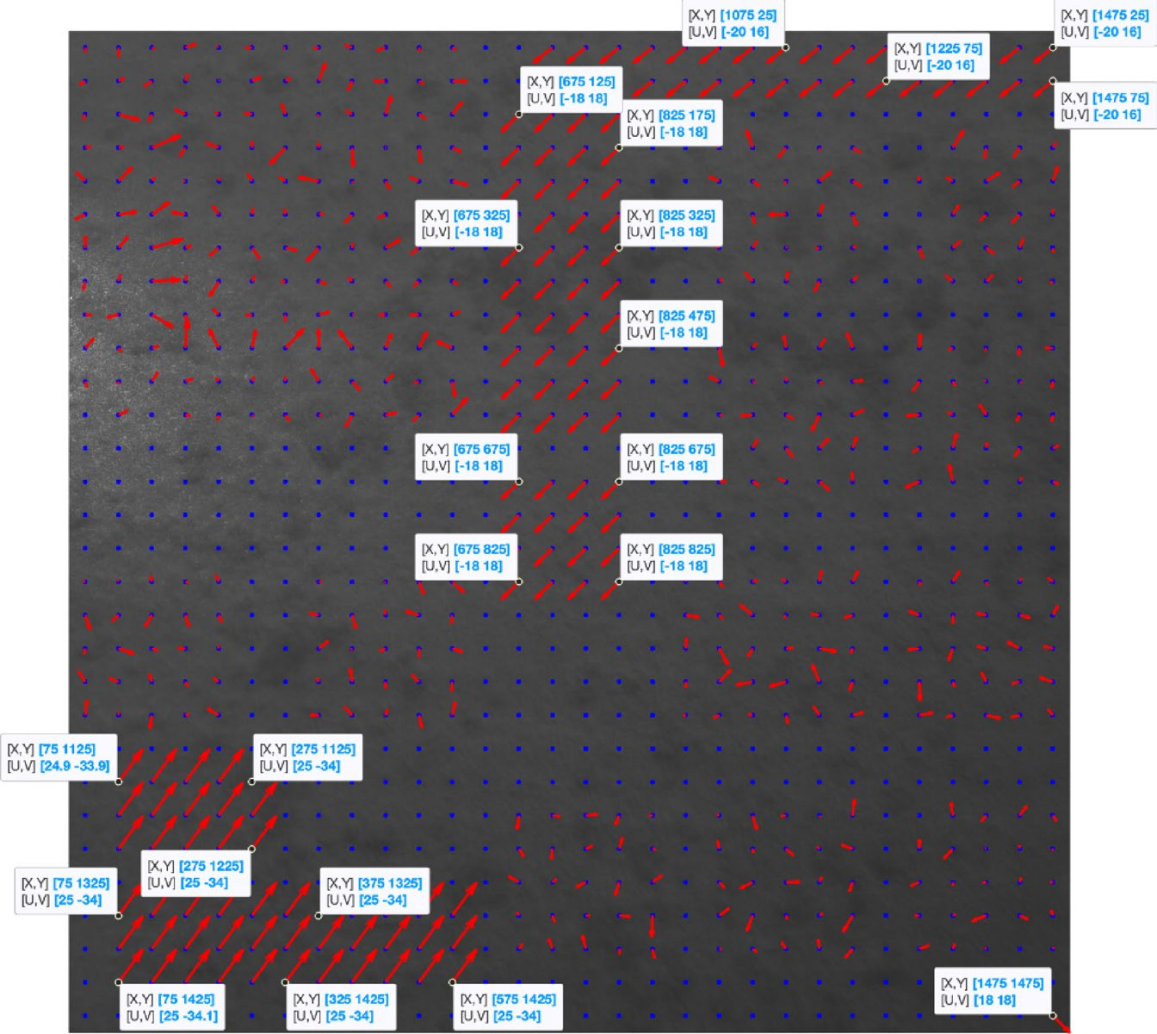
Enlarged Fig. 4a, with the computed displacements highlighted.

In Fig. 5 (Fig. 4a enlarged and detailed), we show some of the computed displacements using an overlap of 50% in the area of the two grids.

Note that in MATLAB, the printed V’s sign is the opposite of the actual shift sign (Y-direction); however, as the arrow plots demonstrate, the calculated direction is correct. As shown in Fig. 5, the computed shifts (U, V) completely match the actual shifts.

**Fig. 6:**
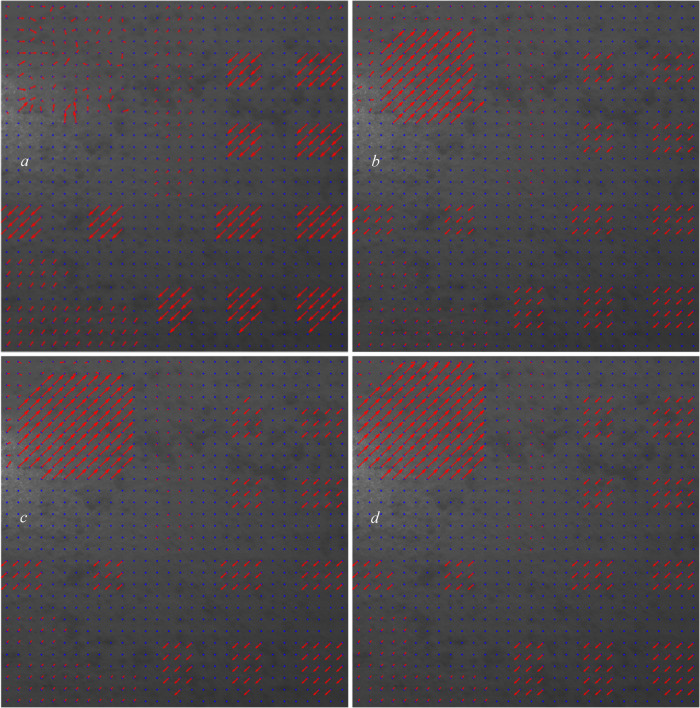
Displacement vectors with the overlaps of (**a**): 2.5, (**b**): 3, (**c**): 3.5, and (**d**): 4.

In Fig. 6, we tested the overlap percentages of (2.5, 3, 3.5, 4) $\:\times\:$100.

Based on the four images plotted in Fig. 6, we can see that the overlap of 3 (Fig. 6b) suits the affected area three very well. However, because with the overlap of 2.5, none of the displacements in the affected area one’s grids are correctly shown, we choose an overlap of 2.5 (Fig. 6a) for the affected area three for training the CNN structure to allow it to have a better distinction between the overlaps of areas one and three.

**Fig. 7:**
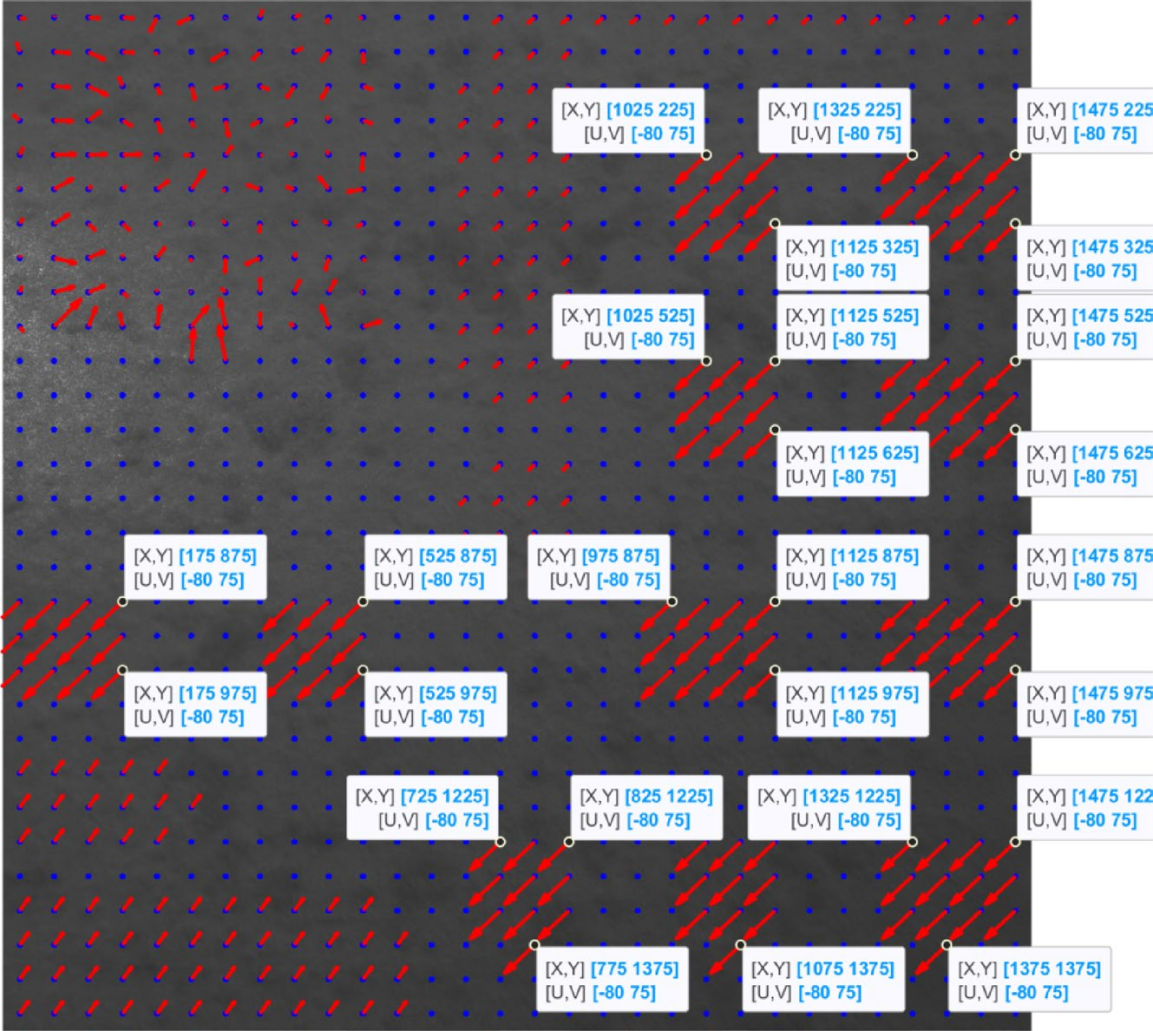
Enlarged Fig. 6a, with the computed displacements highlighted.

In Fig. 7 (Fig. 6a enlarged and detailed), we show some of the computed displacements using an overlap percentage of 250 in area three.

As shown in Fig. 7, the computed shifts (U, V) completely match the actual shifts of 80 pixels to the left and 75 pixels down in area three.

**Fig. 8:**
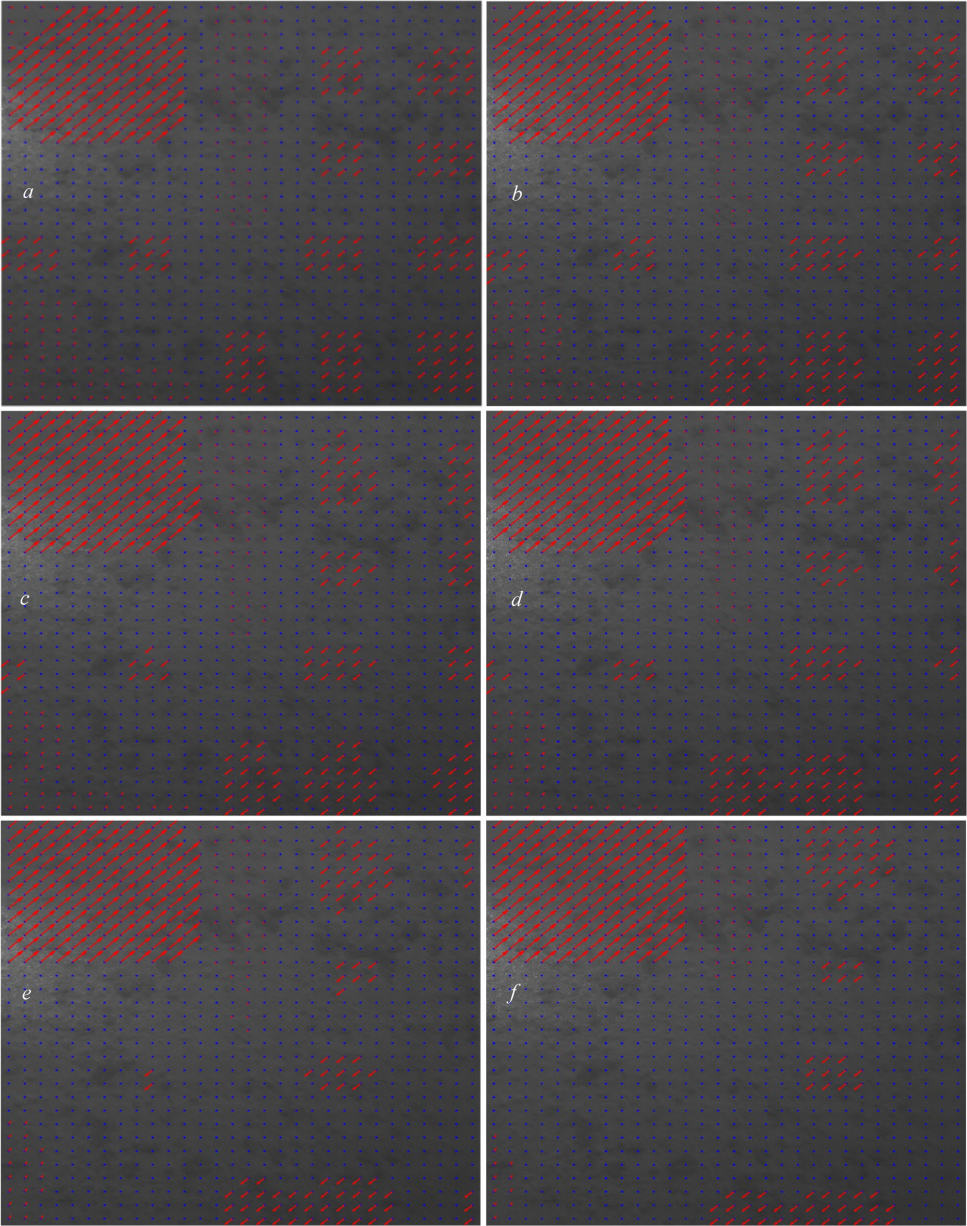
Displacement vectors with the overlaps of (**a**): 4.5, (**b**): 5, (**c**): 5.5, (**d**): 6, (**e**): 7, and (**f**): 8.

In Fig. 8, we test the overlap percentages of (4.5, 5, 5.5,6.7,8) $\:\times\:$100.

**Fig. 9:**
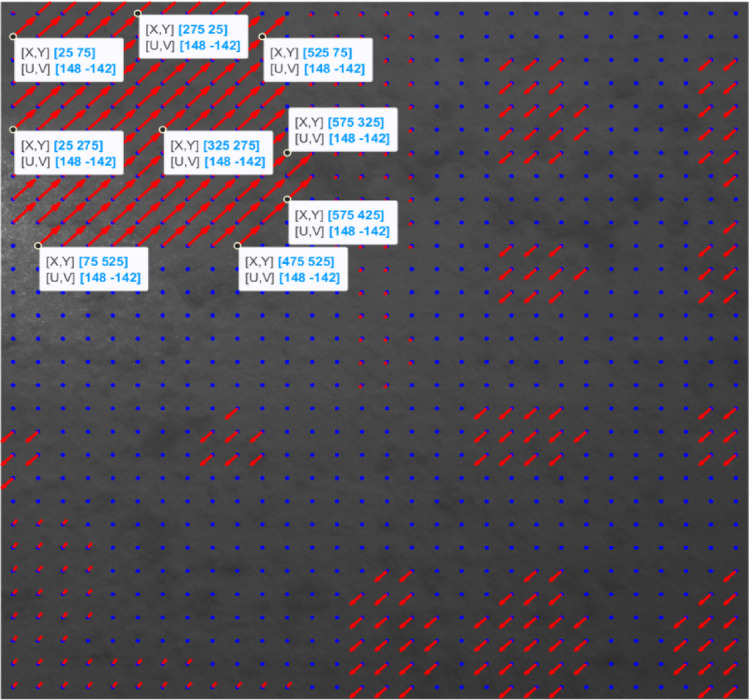
Enlarged Fig. 8c, with the computed displacements highlighted.

Based on the six images plotted in Fig. 8, we can see that the overlap of 8 (Fig. 8f) suits the affected area one very well by covering almost all the grids in this area. However, because the overlap of 8 might cause overfitting and is much larger than the medium overlap of 2.5, in order to have a better-trained structure and make it capable of detecting more diverse displacement cases, we chose an overlap of 5.5 for the area one (due to the onset of this overlap, no erroneous arrows appear to be displaced within the grid area one). In Fig. 9 (Fig. 8c enlarged and detailed), we show a few of the computed displacements using an overlap of 5.5 in the affected area one.

As shown in Fig. 9, the computed shifts (U, V) completely matched the actual shifts in area one.

**Fig. 10:**
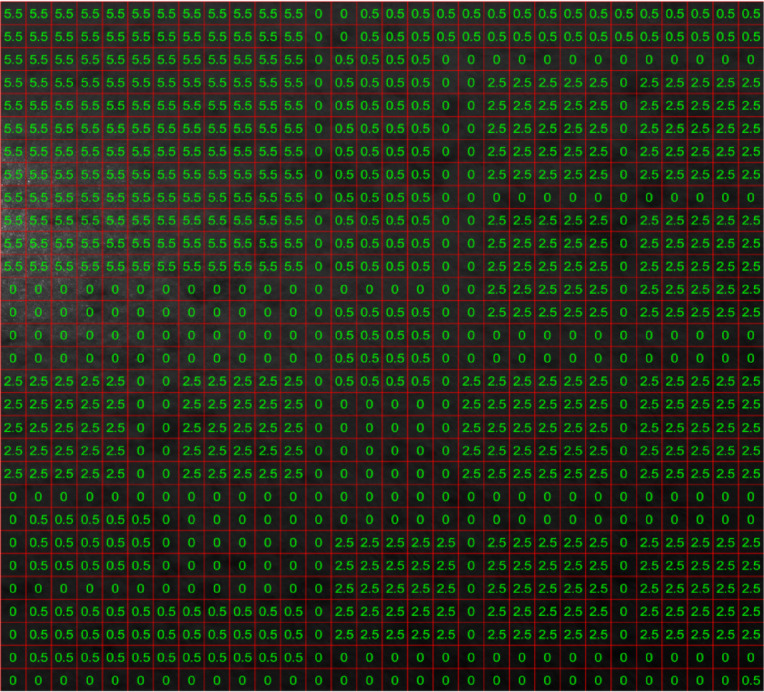
Manually dedicated labels to each grid (sample) of dataset.

Based on the results obtained in Figs. 4, 5, 6, 7, 8 and 9, the labels (overlaps) pattern used for training the CNN structure is shown in Fig. 10.

As can be seen in Fig. 10, the dedication of overlap percentages to each grid is performed based on the actual affected areas shown in Fig. 3 and not necessarily the ones in Figs. 4, 5, 6, 7, 8 and 9. We used four labels (overlaps) in this analysis: 0 (no overlap), 0.5 (small overlap), 2.5 (medium overlap), and 5.5 (large overlap), to cover a large range of affected areas and displacement sizes and enable the CNN-based model to detect and measure any displacements in the speckle pattern.

### Training and testing the CNN model

After creating the dataset and labels (input layer), we trained the CNN model using them. As detailed in Sect. 3, in order to train the CNN model, we first determined the hyperparameter values/type. As mentioned in Sect. 3, the hyperparameters were the learning rate, mini-batch size, number of epochs, number of filters (kernels), dropout rate, weight decay, and optimizer type. We tested three possible (typical) values (small, medium, and large) for each hyperparameter as well as two types for the optimizer, to determine the best hyperparameters set to be used for training the CNN model. These possible values/types are learning rate $\:\in\:$ [10^− 5^, 10^− 3^, 10^− 2^], mini-batch size $\:\in\:$ [16, 32, 64], number of epochs $\:\in\:$ [50, 100, 200], number of filters (kernels) $\:\in\:$ [16, 32, 64], dropout rate $\:\in\:$ [0.1, 0.3, 0.5], weight decay $\:\in\:$ [10^− 4^, 10^− 3^, 10^− 2^], and optimizer type $\:\in\:$ {Adam, SGDM}. As again mentioned in Sect. 3, to choose the best set (combination) of hyperparameter values/type, we use a *k*-folding strategy using a Monte Carlo simulation. We ran the simulation 1000 times to find the best hyperparameter set, which yielded the maximum value of our objective function (Eq. 18). We used five folds in this study. Therefore, in each simulation run, we divided the dataset into five folds, performed a *k*-folding procedure, and obtained a single score (Eq. 17) for the run. Then, we choose the hyperparameter set corresponding to the run with the maximum score among the 1000 runs as our CNN model’s hyperparameters.

**Fig. 11:**
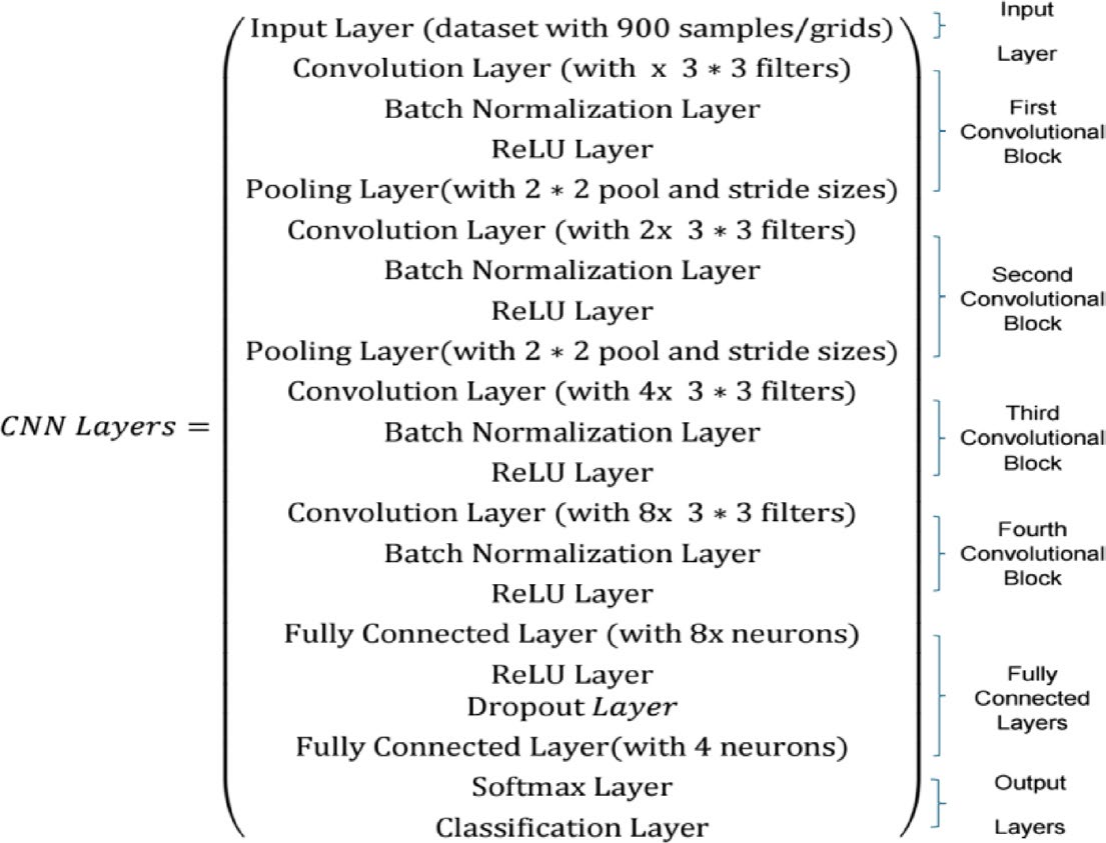
The proposed CNN architecture.

Before training, the number of CNN layers was determined. Different types of layers in a CNN structure are explained in Sect. 3, and we should now determine the number of each layer type, and in which order we want to use in our CNN model. Owing to the nature and complexity of our problem, we chose the structure shown in Fig. 11.

After running the simulation, the best set was obtained as follows: learning rate = 0.001, mini-batch size = 64, number of epochs = 50, x = number of filters (kernels) = 16, dropout rate = 0.5, weight decay = 0.01, and optimizer type = SGDM. While running the simulation and obtaining the hyperparameter values/type, the performance on the validation set was evaluated every 100 iterations, and the training was stopped if the validation performance did not improve for five consecutive validation checks.

**Fig. 12:**
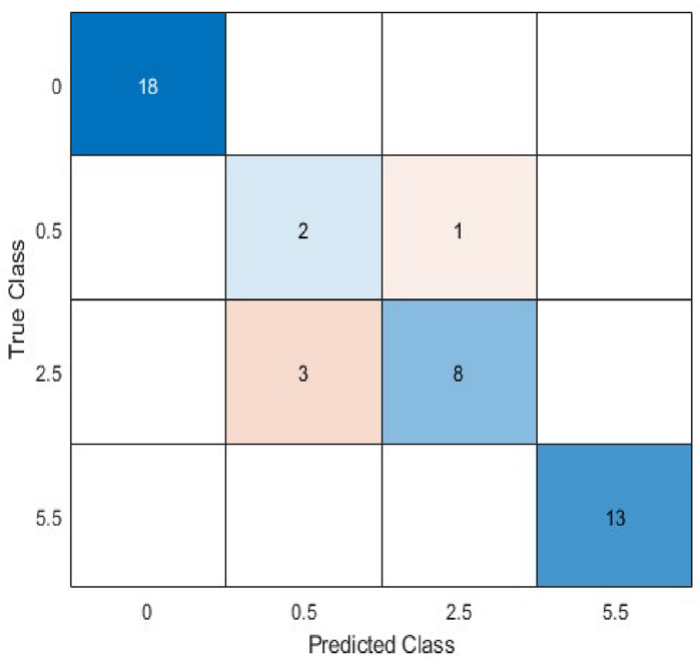
Confusion chart for the final trained model on 5% of the dataset.

After obtaining the values/type for the hyperparameters, we used the obtained set to train the CNN structure. We first divided the dataset into training and test datasets, and because we did not have a large dataset, we use 5% of the samples (45 out of 900 grids) as the test set and 95% (855 out of 900 grids) for training. The confusion chart for the test dataset after training is shown in Fig. 12.

The computed scores based on the confusion chart were weighted Precision = 0.9328, weighted Recall = 0.9111, weighted F1-Score = 0.9178, Accuracy = 0.9111, and the Final Score = 0.5 $\:\times\:$ 0.9178 + 0.5 $\:\times\:$ 0.9111 = 0.9144. Furthermore, to evaluate the sensitivity of the model’s performance to the train-test split ratio, we also tested two additional splitting scenarios using the same optimal hyperparameters. Using a conventional 80/20 split, the model achieved a weighted F1-score of 0.8846 and an accuracy of 0.8889. This demonstrates that the framework remains highly effective and generalizes well even when evaluated on a larger, more robust test set. Conversely, as expected, model performance improved with a 97.5/2.5 split, yielding a weighted F1-score of 0.9351 and an accuracy of 0.9545, which is attributable to the larger volume of training data available. This analysis confirms that while a larger training set enhances performance, the model’s effectiveness is consistent and reliable across different data partitioning strategies.

Now, we apply the trained CNN model to the shifted case in Sect. 4.1 (Fig. 2b) to determine if it can detect and measure displacements. The results are as follows:

**Fig. 13:**
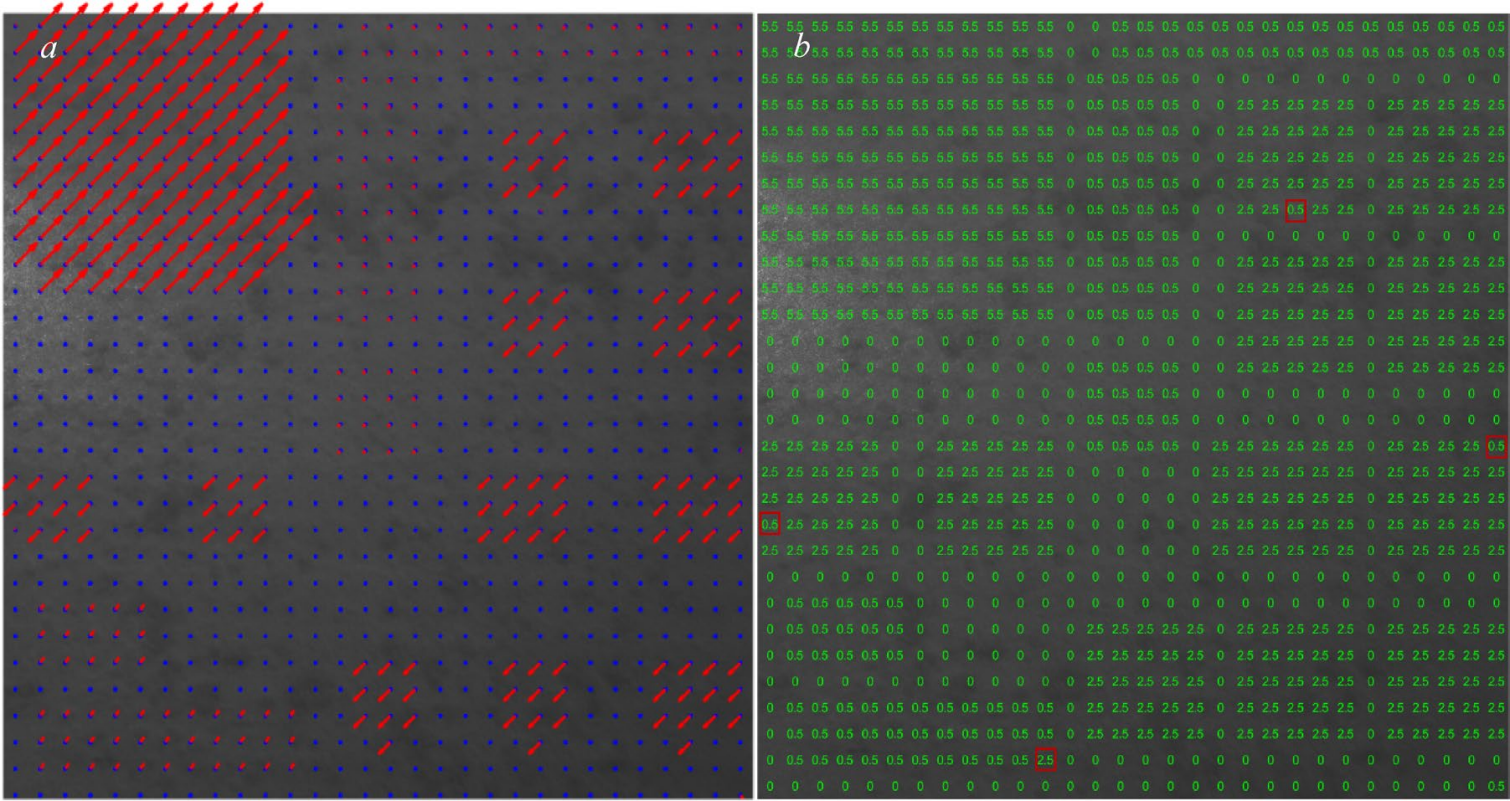
Testing the CNN model on the data set it was trained with (**a**): displacement arrows and (**b**): dedicated overlaps.

As can be seen in Fig. 13, the CNN model perfectly performs what it is supposed to do, and its output is a perfect combination of Fig. 4a (for area two), 6*a* (for area three), and 8*c* (for area one). In addition, the dedicated overlap percentages using the CNN model in Fig. 13b mostly matches the manual assignments used for training shown in Fig. 10, with only four grids out of 900 grids (marked with red in Fig. 13b) wrongly dedicated.

We also tested the CNN model in another simulated case. We again made artificial shifts on the reference image (Fig. 2a), and this time the sizes of the affected areas and the shift sizes for each area of the shifted image are as follows:

Upper-Left Corner:

- Shift in the X direction: 74 pixels to the right.
- Shift in the Y direction: 90 pixels up.
- Affected area size: 200 pixels (width) × 300 pixels (height).

Upper-Right side of the image, but not in the corner; after an area of 300 × 300 pixels:

- Shift in the X direction: 28 pixels to the left.
- Shift in the Y direction: 10 pixels down.
- Affected area size: 200 pixels (width) × 200 pixels (height).

Bottom-Right Corner:

- Shift in the X direction: 8 pixels to the right.
- Shift in the Y direction: 8 pixels down.
- Affected area size: 90 pixels (width) × 90 pixels (height).

Bottom-Left side of the image, but not in the corner; after an area of 150 × 150 pixels:

- Shift in the X direction: 20 pixels to the right.
- Shift in the Y direction: 34 pixels up.
- Affected area size: 150 pixels (width) × 150 pixels (height).

Center:

- Shift in the X direction: 25 pixels to the right.
- Shift in the Y direction: 18 pixels down.
- Affected area size: 250 pixels (width) × 250 pixels (height).

Middle-Right side:

- Shift in the X direction: 33 pixels to the left.
- Shift in the Y direction: 35 pixels up.
- Affected area size: 200 pixels (width) × 200 pixels (height).

First, we ran the image registration algorithm in Sect. 2 (without overlaps) to detect the affected areas. The results are shown in Fig. 13.

**Fig. 14:**
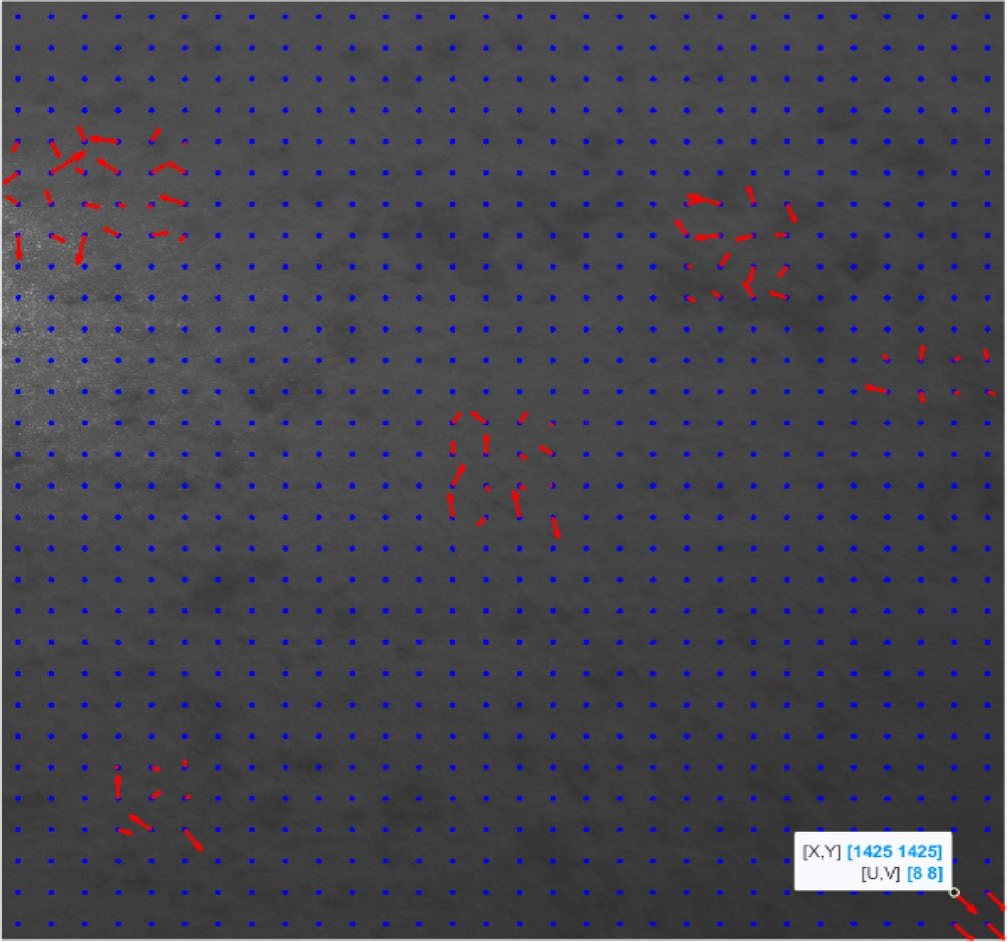
Testing the CNN model on another simulated case; with no overlaps.

Based on the results shown in Figure 14, the displacement size for the bottom right area is so small that no overlaps are needed for its measurement.

**Fig. 15:**
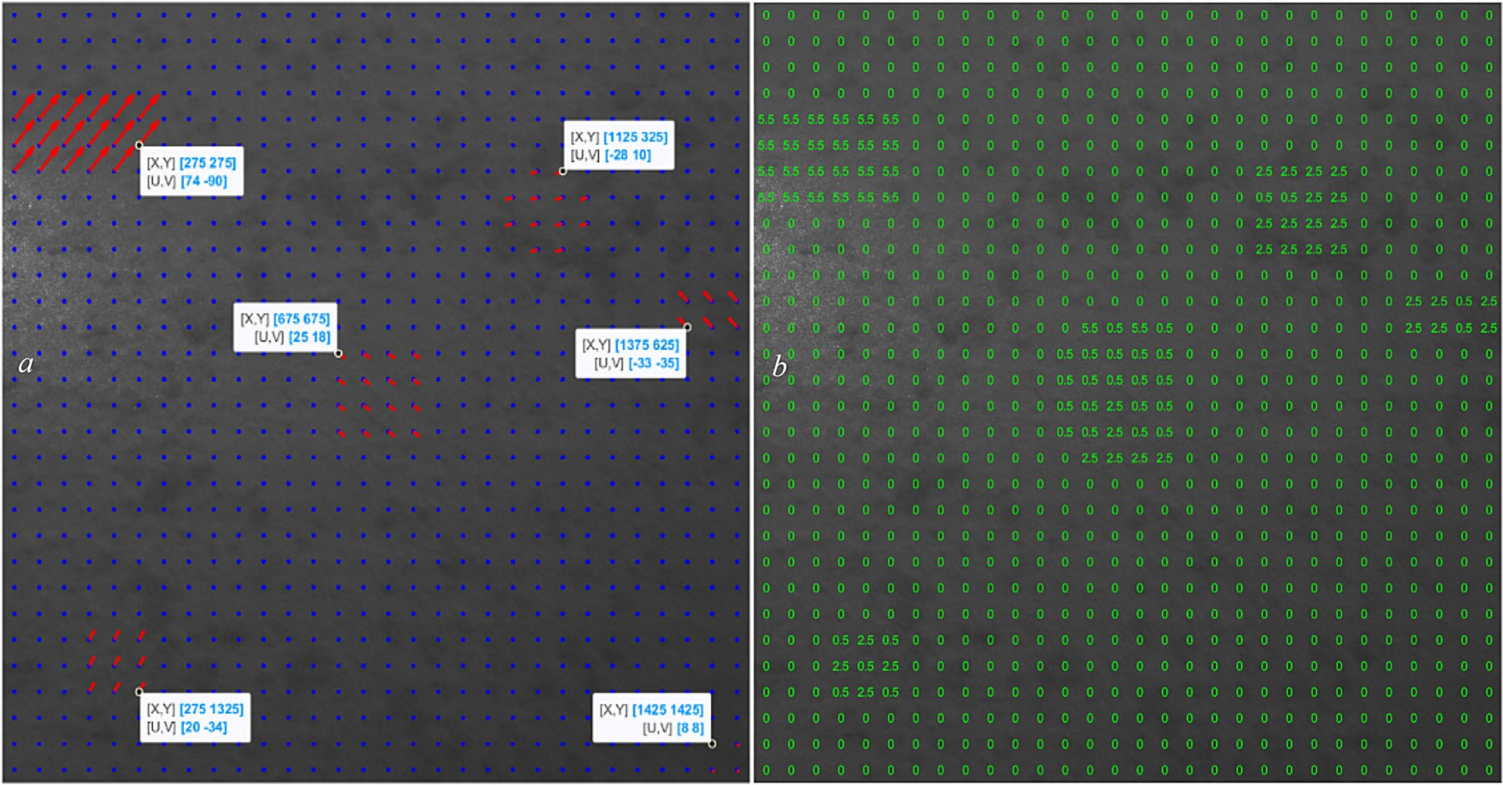
Testing the CNN model on another simulated case; using CNN’s intelligent overlap assigning, with computed displacement arrows (**a**) and the dedicated overlaps to each grid (**b**).

After running the CNN model on the image and letting it automatically assign the overlap percentages for each grid individually, the displacement sizes for each grid were calculated, and the results are as follows. The dedicated overlaps of each grid by the CNN model are also shown in Fig. 15b.

As shown in Fig. 15, the algorithm performed a great job in both detecting the affected areas and correctly measuring the displacements. The accurate detection and measurement of these previously unseen displacements demonstrate the model’s strong generalization capability and its competency to handle a wide range of displacement magnitudes and patterns beyond its specific training examples.

### Applying on the real case

Now, we return to the original case introduced at the beginning of Sect. 4. An experimental setup was used to capture a reference speckle pattern image from a laser-illuminated and water-sprayed cardboard surface. We then created some artificial shifts on the reference speckle pattern image and used it as our second (shifted) image and trained our CNN model using these two images in Sect. 4.2. Subsequently, we tested the CNN model on another artificially shifted image. In this section, we will test the trained model on the real case, we first set up.

**Fig. 16:**
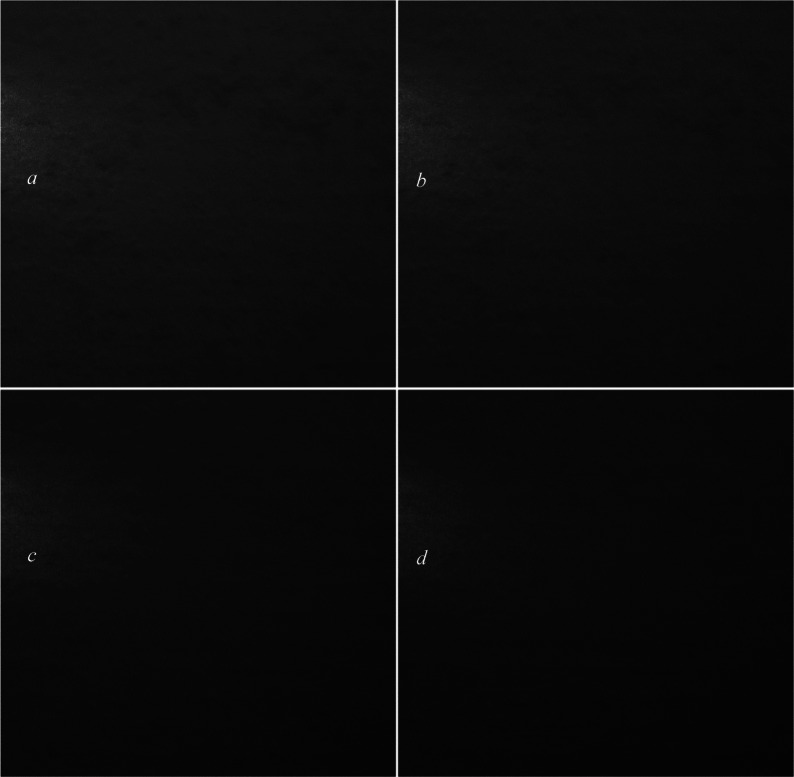
Speckle patterns of the images taken from the water-sprayed cardboard 15 s after the reference image (**a**), 30 s after the reference image (**b**), 45 s after the reference image (**c**), and 60 s after the reference image (**d**).

In addition to the reference image captured after spraying water, we also took four more images of the cardboard at 15-second intervals. The resulting speckle patterns are shown in Figure 16.

As shown in Fig. 16, the differences between these speckle patterns and the reference speckle pattern in Fig. 2a are too subtle to be detected by the naked eye.

**Fig. 17:**
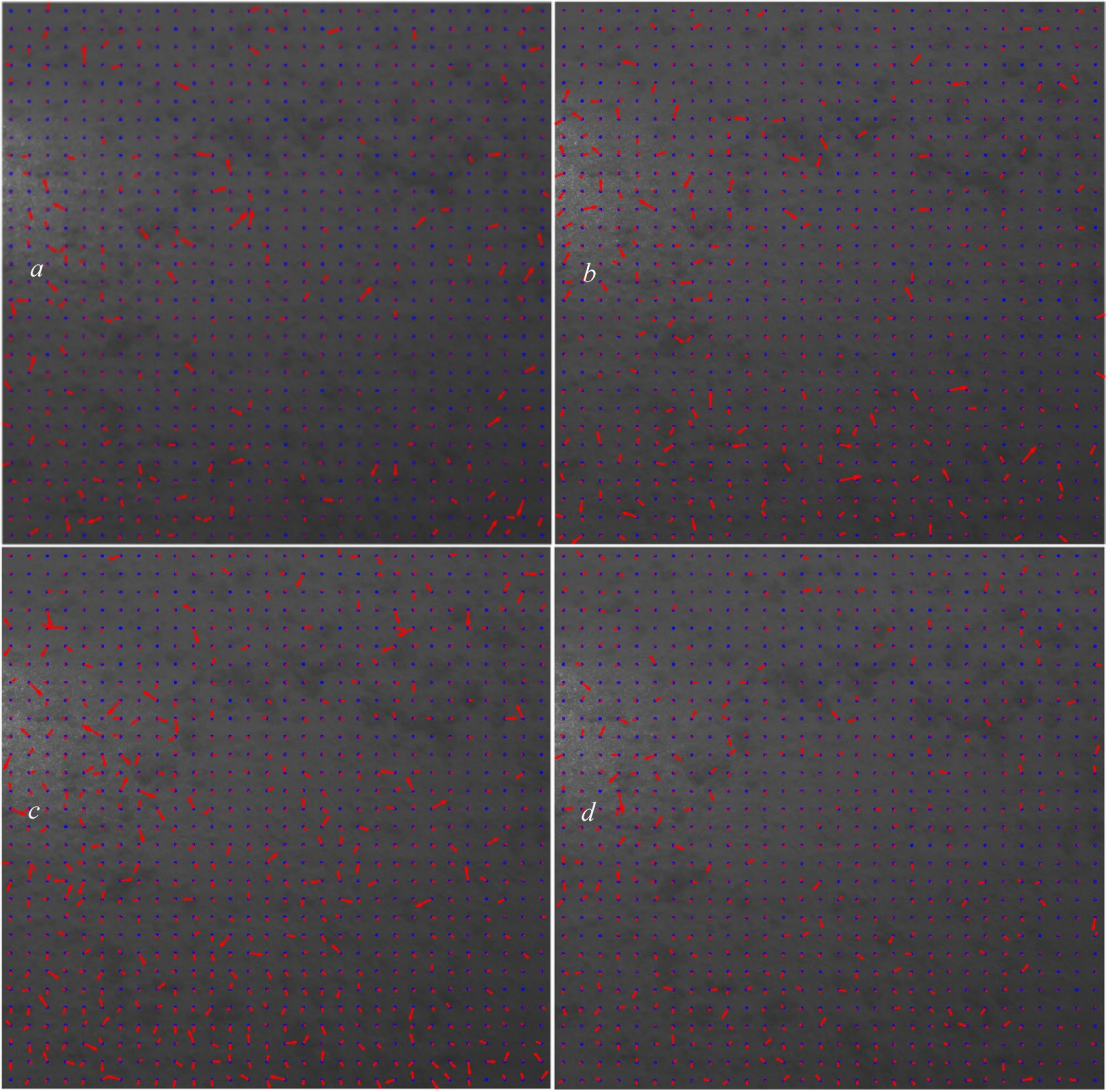
The affected areas of the reference image with drawn displacement arrows relative to the pictures taken 10 s after the reference image (**a**), 20 s after the reference image (**b**), 30 s after the reference image (**c**), and 40 s after the reference image (**d**).

Next, we used the algorithm described in Sect. 2 (with no overlaps) to detect the affected areas. The results for all four speckle patterns are shown in Fig. 17.

As shown in Fig. 17, all the grids of the images show displacements, and the algorithm described in Sect. 2 successfully detected displacements in all grids of the four images. However, without grid overlap, the detected speckle shifts appeared chaotic and lacked a coherent direction. Ideally, the overall speckle displacements should exhibit a consistent downward trend owing gravity or follow the direction of water penetration. The absence of overlap contributes to this irregularity, and additional factors such as uneven water distribution and surface inconsistencies also affect the results. Consequently, the drawn arrows in this scenario are unreliable and should be interpreted with caution.

**Fig. 18:**
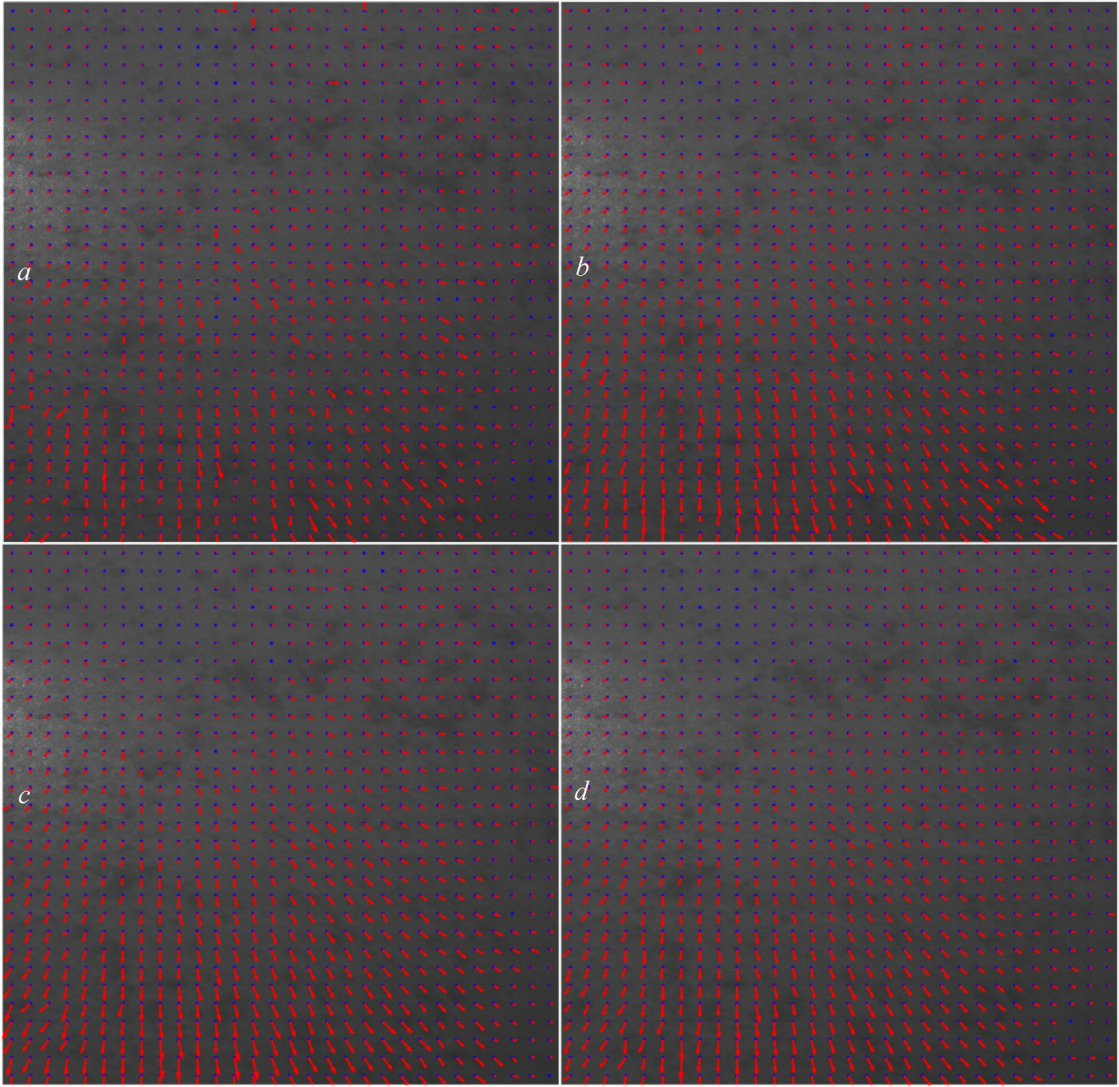
The displacement arrows relative to the reference image, computed using the intelligent CNN-based overlap dedication mechanism for the pictures taken 15 s after the reference image (**a**), 30 s after the reference image (**b**), 45 s after the reference image (**c**), and 60 s after the reference image (**d**).

To correctly measure and show the displacement arrows, we used our intelligent CNN-based overlap dedication mechanism, detailed in Sect. 3, and trained in Sect. 4.2. The results are shown in Figure 18.

As can be seen, most of the arrows in Fig. 18 move downward, which is what we expected gravity does for the water drops on the surface of the cardboard. In addition, because the surface of the cardboard is not completely smooth or level, some movements in other directions are visible and normal.

**Fig. 19:**
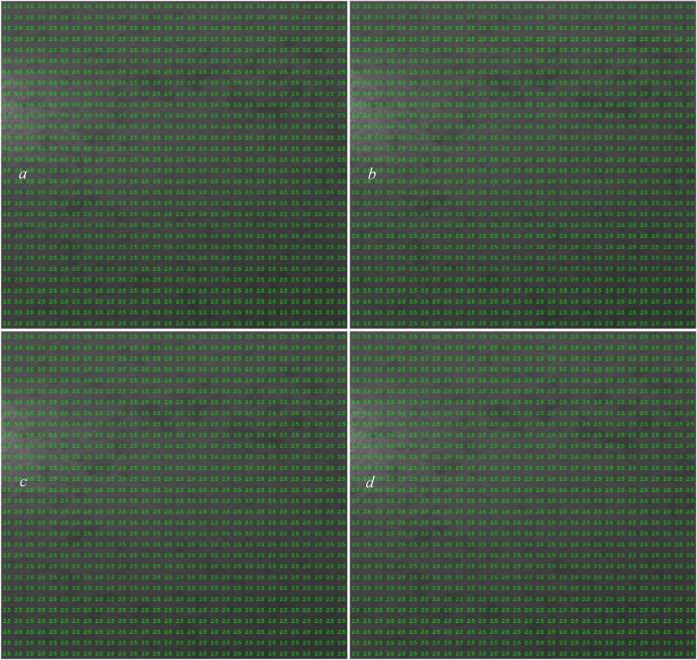
The dedicated overlap percentages to each grid of the images taken 15 seconds after the reference image (**a**), 30 seconds after the reference image (**b**), 45 seconds after the reference image (**c**), and 60 seconds after the reference image (**d**).

Figure 19 shows the dedicated overlap percentages for each grid of each image using the CNN model. As is clear, the overlap percentage of 2.5 $\:\times\:100$ (250%) is dedicated to most of the grids by the CNN framework.

This setup effectively demonstrates the use of laser speckle imaging and the performance of the proposed algorithm in tracking micro-dynamic changes on a surface. The speckle displacement tracking algorithm is a powerful tool for measuring the temporal evolution of surface changes, such as those caused by liquid absorption.

## Concluding remarks

In this study, a novel AI-based framework was developed to perform both detection and measurement of speckle displacements, in two main steps. In the first step, a Fourier-based registration mathematical framework was used, in which the speckle pattern was divided into equal-sized grids and alignment was applied to each grid individually. This enabled us to detect simultaneous speckle displacements of different sizes in different areas of the speckle pattern, provided that the grid sizes were sufficiently small. However, this procedure was only able to correctly measure displacements, if they were too small. In addition, shifts could not be detected if the grid size was noticeably larger than the size of the affected area. To overcome these issues, overlapping of the grids (grids in the reference image and the grids in the deformed image) was considered. Nonetheless, determining a suitable overlap size proved challenging, especially in real cases where the nature of the displacements is unknown (both the displacement size and the affected area). Determining the overlap sizes depends on the grid size, size of the affected (deformed) areas, and displacement sizes. Therefore, we developed a convolutional neural network (CNN) model to intelligently dedicate overlap percentage values to each grid of the image individually. We optimized the CNN’s hyperparameter values/type using a Monte Carlo simulation-based algorithm with *k*-folding cross validation. To test our method, we presented an experimental optical setup in which speckles displacements were measured on a water-sprayed cardboard. The first image captured after water spraying was used as the reference image. Then, we applied artificial shifts to the reference image and created a deformed image to conduct a preliminary analysis and train the CNN model. We then applied our developed method to four other images captured a short time apart after the water spray and compared them to the reference image (the first image taken after the water spray). The results showed the effectiveness of the proposed framework in both the detection and measurement of displacements in simulated and real cases.

The current framework is designed for in-plane translational displacements. Out-of-plane motion or large rotations can decorrelate speckle patterns and challenge the method. The CNN also requires a pre-labeled dataset for training, which can be time-consuming and challenging to create. Future directions can include extending the framework to handle rotational and out-of-plane displacements, developing unsupervised or semi-supervised learning techniques to reduce the reliance on manual labelling, and exploring real-time implementation for industrial inline inspection. To further strengthen the validation of our method, future work can also focus on applying this framework to real-world scenarios with known ground-truth displacements, such as controlled mechanical testing, to enable precise quantitative error analysis.

## Data Availability

Data used in this study will be available upon request from the corresponding author.
